# Toad venom-derived bufadienolides and their therapeutic application in prostate cancers: Current status and future directions

**DOI:** 10.3389/fchem.2023.1137547

**Published:** 2023-03-16

**Authors:** Qingmei Ye, Xin Zhou, Fangxuan Han, Caijuan Zheng

**Affiliations:** ^1^ Key Laboratory of Tropical Medicinal Resource Chemistry of Ministry of Education, Key Laboratory of Tropical Medicinal Plant Chemistry of Hainan Province, College of Chemistry and Chemical Engineering, Hainan Normal University, Haikou, Hainan, China; ^2^ Hainan General Hospital & Hainan Affiliated Hospital of Hainan Medical University, Haikou, Hainan, China; ^3^ The Fifth People’s Hospital of Hainan Province & Affiliated Dermatology Hospital of Hainan Medical University, Haikou, Hainan, China

**Keywords:** toad venom-derived bufadienolides, therapeutic application, prostate cancers, current status, future directions

## Abstract

Cancer is the second leading cause of death worldwide. Specially, the high incidence rate and prevalence of drug resistance have rendered prostate cancer (PCa) a great threat to men’s health. Novel modalities with different structures or mechanisms are in urgent need to overcome these two challenges. Traditional Chinese medicine toad venom-derived agents (TVAs) have shown to possess versatile bioactivities in treating certain diseases including PCa. In this work, we attempted to have an overview of bufadienolides, the major bioactive components in TVAs, in the treatment of PCa in the past decade, including their derivatives developed by medicinal chemists to antagonize certain drawbacks of bufadienolides such as innate toxic effect to normal cells. Generally, bufadienolides can effectively induce apoptosis and suppress PCa cells *in-vitro* and *in-vivo*, majorly mediated by regulating certain microRNAs/long non-coding RNAs, or by modulating key pro-survival and pro-metastasis players in PCa. Importantly, critical obstacles and challenges using TVAs will be discussed and possible solutions and future perspectives will also be presented in this review. Further in-depth studies are clearly needed to decipher the mechanisms, e.g., targets and pathways, toxic effects and fully reveal their application. The information collected in this work may help evoke more effects in developing bufadienolides as therapeutic agents in PCa.

## 1 Introduction

The quality of life of cancer patients have been improved significantly due to the progress of application of new technologies, including drug development, especially precision medicine. Targeted therapies and the cutting-edge immunotherapies have reached a new paradigm for cancer treatment, which work together with renovated surgery and radiotherapy, etc., to markedly improve treatment outcomes. However, there are still many challenges in treating certain types of cancer including prostate cancer (PCa) which has two unique characteristics. The first one is the high prevalence since it’s one of the leading cancers in men and one of the leading causes of deaths among men worldwide (Gomella, 2017; Schatten, 2018; Zhang et al., 2022a). The second characteristic is the high incidence of drug resistance, since more than 90% of PCa will eventually develop resistance to androgen-depredation therapy (ADT), termed as castration-resistant PCa (CRPC), and later second resistance to subsequent chemotherapies (Armstrong and Gao, 2015; Cohen et al., 2021; Liotti et al., 2021; Morel et al., 2021; Ji et al., 2022; Peery et al., 2022). It’s known that various factors contribute to the development of drug resistance in PCa, such as the alteration/mutation of androgen receptor (AR) or oncogenes, metabolism adaptation, overexpression of ATP-binding cassette (ABC) transporters, apoptosis resistance, enhanced DNA repair and cellular defensive systems against toxic inducers, etc. (Peery et al., 2017; Wang et al., 2020a; de Leeuw et al., 2020; Messina et al., 2020; Peery et al., 2020; Yang et al., 2020; Do and Webster, 2021; Filon et al., 2022). Thus, structurally and mechanistically renovated agents that can effectively suppress PCa and/or less likely develop resistance are in urgent need.

**FIGURE 1:**
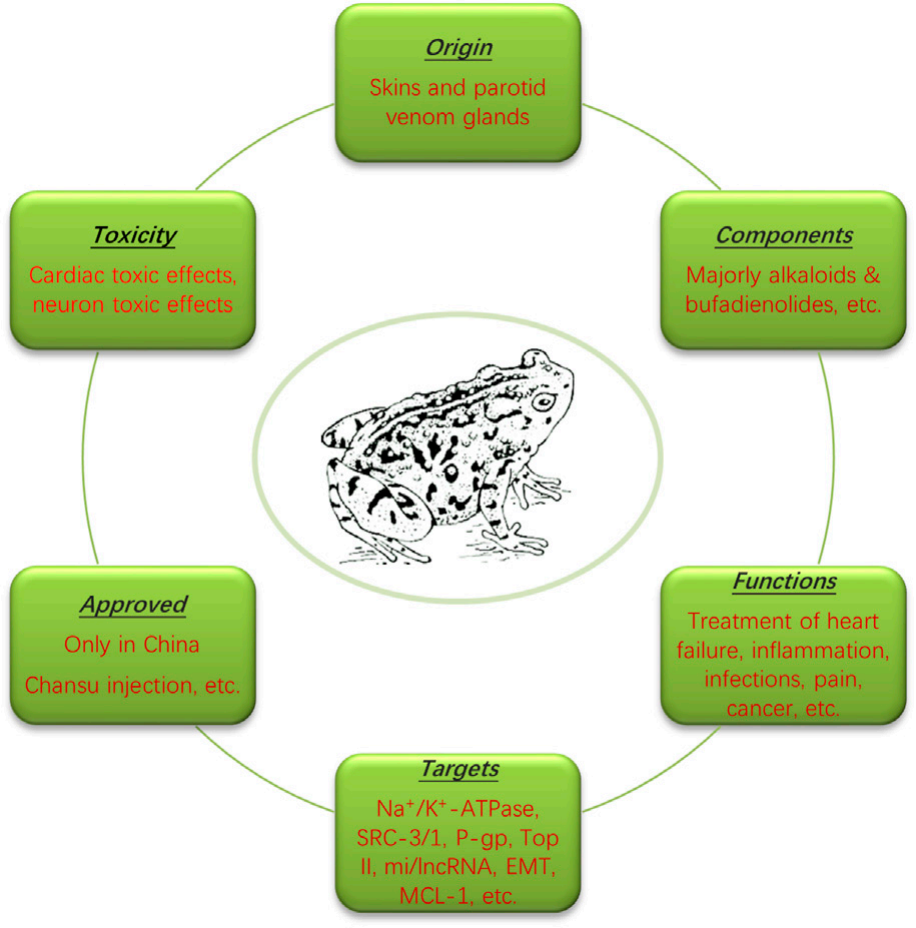
The origin, components, functions, toxicity and mechanisms of toad venom. Originated from the skin and auricular glands of Chinese toad, toad venom contains mostly alkaloids and bufadienolides, functioning to treat heart disease, inflammation, infection, pain and cancer through regulating Na^+^/K^+^-ATPase, SRC-3 and -1, etc. Drugs containing toad venom are only approved in China.

Toad venom, also named as Chan-Su, is a traditional Chinese medicine that has shown therapeutic efficacies in clinic (mainly in China) and has been widely used for the treatment of cancer, cardiovascular diseases, pain, and inflammation/inflammatory diseases as shown in Figure 1 (Gao et al., 2017; Li et al., 2021a; Xu et al., 2021; Zheng et al., 2022). Originally derived from the skin and auricular glands of Chinese toad, toad venom is used to repel toad’s natural enemies primarily, working as a protective agent. Known for the toxic effects to cause cardiac arrhythmia, toad venom-derived agents (TVAs) usually work as an inhibitor of Na^+^/K^+^-ATPase and a regulator of calcium homeostasis, which leads to seizure and coma, etc., thereby causing toxic effects (Chen and Kovarikova, 1967; Bick et al., 2002; Lopez-Lopez et al., 2008). In addition to toxic effects, however, toad venom has therapeutic effects that can be applied to treat certain diseases. Till now, due to its strict export ban to other countries by state law, drugs that contain toad venom are only approved for clinical use in China, such as Chansu injection, Liu Shen Wan, Xin Bao Wan, Chan-Su Wan, Hua-Chan Wan (made of isolated cinobufagin in toad venom), Kyushin, Zuo Xiang Bao Xin Wan, etc. (Morishita et al., 1992). In addition to Chinese Chan-Su in the application of cancer treatments, toad venom from other species has also been reported, including Indian toad venom (Gomes et al., 2011), although they have not been fully studied for its application.

In this review, we focused on the applications of bufadienolides, especially those isolated pure compounds in TVAs, in treating PCa in the past 10 years. While it is true that not too many studies have been published as of December 2022, and that the research and application of TVAs in cancer treatment are still at its early stage, the information collected could certainly serve as a base for their further exploration in PCa treatment.

## 2 Bufadienolides in toad venom and their therapeutic implication in cancers

**FIGURE 2:**
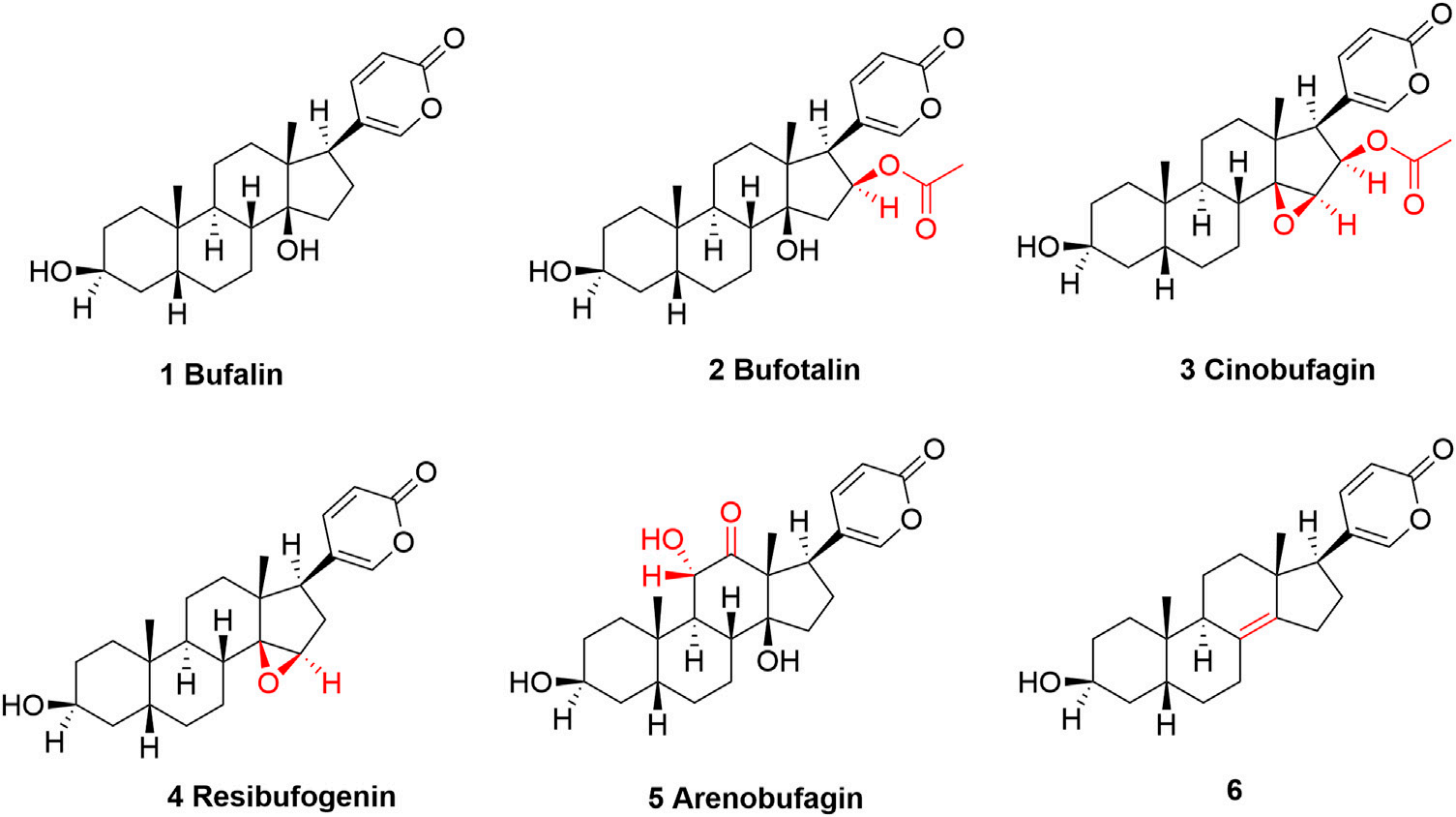
The structures of bufalin and its derivatives found in toad venom which show cancer-suppressing effects. Structurally, bufalin can be regarded as a parent compound, and all other TVAs are modified on bufalin at different positions. The structural differences are highlighted in red.

In total, several dozens of different components were identified and characterized in toad venom (Zhang et al., 2005; Wang et al., 2018a; Cao et al., 2019). Their pharmacological effects can be majorly attributed to alkaloids (Dai et al., 2018a; Dong et al., 2022) and bufadienolides which share steroids scaffold in common (Qu et al., 2012). Both alkaloids and bufadienolides are among the most prominent and most-studied compounds in toad venom. Growing studies have confirmed that both alkaloids and bufadienolides can work in treating cardiovascular diseases and cancers (Chen et al., 2020). Our special and major interest in this review falls in these bufadienolides (Figure 2).

There are several prominent members that are categorized as bufadienolides (Figure 2) (Cunha-Filho et al., 2010; Zhang et al., 2014), including Bufalin, (3β,14-dihydroxy-5β-bufa-20,22-dienolide, shown as compound **1**) (Zhang et al., 2020a), bufatalin [(3β,14,16β-Trihydroxy-5β-bufa-20,22-dienolide) 16-acetate, shown as compound **2**] (Zhang et al., 2022b), cinobufagin, (3β-Hydroxy-14,15β-epoxy-5β-bufa-20,22-dienolid-16β-yl acetate, shown as **3**) (Toma et al., 1987), resibufogenin [(3β,5β,15β)-14,15-epoxy-3-hydroxy-bufa-20,22-dienolide, shown as **4**] (Yang et al., 2021a), and arenobufagin [(3β,5β,11α)-3,11,14-Trihydroxy-12-oxobufa-20,22-dienolide, shown as **5**] (Zhang et al., 2013). These compounds are structurally related, and specially, all of them can be regarded as bufalin’s derivatives, with minor differences at certain position, which are shown and highlighted in Figure 2. It appears that TVAs have broad-spectrum anticancer potential (Liu et al., 2019; Niu et al., 2021; Jia et al., 2022). Research has indicated that they, either by single use or as a mixture when combined with other agents, are effective in treating acute myeloid leukemia (Hirasaki et al., 2022), lung cancer (Xie et al., 2018; Li et al., 2021b; Meng et al., 2021), colorectal cancer (Li et al., 2019; Bai et al., 2021; Meng et al., 2021), liver cancer (Zhang et al., 2012; Zhao et al., 2019; Zhang et al., 2020b; Yang et al., 2021b), breast cancer (Zhu et al., 2018), oral cancer (Jo et al., 2021), gastric cancer (Xiong et al., 2019), Ehrlich ascites carcinoma (Giri et al., 2018), melanoma (Pan et al., 2019; Zhang et al., 2020c; Kim et al., 2020), nasopharyngeal carcinoma (Pan et al., 2020; Hou et al., 2022), osteosarcoma (Cao et al., 2017; Dai et al., 2018b; Zhang et al., 2019a), cholangiocarcinoma (Ren et al., 2019), myeloma (Baek et al., 2015), etc.

## 3 Therapeutic application of bufadienolides in PCa

### 3.1 Mono-therapy of TVAs in PCa

Bufalin is one of the most intensively studied compounds among all TVAs (Wang et al., 2018b; Lan et al., 2019; Soumoy et al., 2022). A study by Zhang et al. (2018) showed that bufalin worked as an anticancer agent *via* a p53-mediated mechanism in PCa cells both *in-vitro* and *in-vivo*. In p53-mutant DU145 cells and p53-wild type LNCaP cells, bufalin (5–100 nM, 48 h) treatment could upregulate the expression of cleaved poly (ADP-ribose) polymerase (PARP), and downregulate steroid receptor co-activator 1/3 (SRC1/3), AR and prostate specific antigen (PSA). This study showed that bufalin increased p53 expression in LNCaP cells, but decreased p53 in DU145 cells, however, cleaved PARP or p53 was not observed in p53-null PC-3 cells although inhibited proliferation was identified, suggesting a p53-mediated efficacy (Zhang et al., 2018). The microarray detection of certain mRNA levels indicated that in LNCaP cells, bufalin treatment increased p53 and its transcriptional target P21CIP1, as well as mRNAs related to cellular stress and DNA damage response, and certain senescence-associated genes, such as *CYR61/CCNI*, *CTGF/CCN2* and *CDKN1A*, which were then been validated by the subsequent assays of cell cycle distribution (sub G_0/1_) and the presence of senescence-like phenotype (Zhang et al., 2018). The knockdown of p53 could attenuate bufalin-induced apoptosis as indicated by the decreased level of cleaved PARP. Finally, in the *in-vivo* model of LNCaP xenograft, bufalin (1.5 mg/kg body weight, IP, daily) for 9 weeks inhibited tumor growth, resulting in a 67% decrease as compared to untreated group, without affecting body weight significantly which might suggest a safe profile. More importantly, in bufalin-treated tumors, phospho-p53 was increased, confirming the on-target effect and a network of bufalin with p53 (Zhang et al., 2018).

A recent study by Zhang et al. (2019) found that bufalin can alter the expression of both microRNAs (miRNAs) and long non-coding RNAs (lncRNAs) that are critical for PCa (Zhang et al., 2019b). In CRPC DU145 and PC-3 cells, bufalin suppressed the cell viability in a dose-dependent manner, with an IC_50_ value of 0.89 and 1.28 μM, respectively. At lower than the corresponding IC_50_ (to be more specific, at half of the corresponding IC_50_), bufalin could significantly reduce the migration and invasion of DU145 and PC-3 cells as confirmed by the wound healing assay and transwell assay. The authors screened lncRNA alteration after bufalin treatment (0.1–5 µM) using a lncRNA microarray, and they identified that HOX transcript antisense RNA (HOTAIR) was one of the mostly reduced (Zhang et al., 2019b). HOTAIR targets and inhibits miR-520b as confirmed by RNA immunoprecipitation assay; meanwhile, miR-520b can negatively regulate the expression of fibroblast growth factor receptor 1 protein (FGFR1) which plays a pivotal role in PCa progression and metastasis (Yang et al., 2013; Wang et al., 2019). The authors also investigated and confirmed the positive correlation of HOTAIR and FGFR1 with PCa bone metastasis, and that the overexpression of HOTAIR could reverse bufalin-induced cancer-suppressing effects. Thus, this study indicated that bufalin can inhibit PCa proliferation, migration and invasion *via* regulating the HOTAIR-miR520b-FGFR1 loop (Zhang et al., 2019b).

MiRNA-181, composed with subunits miRNA-181a and b, targets apoptosis-associated proteins such as Bcl-2 family members, functioning as a tumor suppressor (Liu et al., 2017; Pei et al., 2020). Zhai et al. (2013) found that in bufalin (10 μM, 24 h)-treated PC-3 cells, miRNA-181a, but not the others such as miRNA-10b, −17, 18a, 20a, 21, −106, −155, −221 and −372, was markedly upregulated (5-fold), which was later confirmed to be a dose-dependent manner (1, 10 and 15 µM) (Zhai et al., 2013). In PC-3 cells, bufalin (15 µM) significantly decreased the expression of Bcl-2, an anti-apoptotic protein, accompanied with caspase-3 protein activation (*via* testing the level of cleaved caspase-3), which is essential in promoting apoptosis (Kesavardhana et al., 2020). At the same time, the rescue experiments showed that bufalin-induced apoptosis and caspase-3 proteins activation can be partially reversed by miR-181a inhibitor co-treatment (100 nM), validating the targeted effects of bufalin toward miR-181a (Zhai et al., 2013).

Structurally, bufalin is a hydrophobic compound that may encounter poor absorption and bioavailability (Shao et al., 2021). Thus, Liu and Huang (2016) constructed an amphiphilic targeting brush-type copolymers that can deliver bufalin to CRPC cells, which exhibited controlled drug release and higher anticancer capability than free bufalin both *in-vitro* and *in-vivo* (Liu and Huang, 2016). This constructed BUF-loaded micellar nanoparticle BUF-NP-(G3-C12) was found to have an IC_50_ value of 8.0 ng/mL, which was lower than that of free bufalin (which was 13.3 ng/mL) in CRPC DU145 cells; and consistent results were also observed in inducing apoptosis. In DU145 xenograft model, when used by intravenous injection iv) at an equivalent 1.0 mg bufalin/kg, BUF-NP-(G3-C12) showed significantly higher tumor-inhibiting effects than that of free bufalin. Importantly, it didn’t change body weight as compared to vehicle control, suggesting its safety (Liu and Huang, 2016). Further evaluation is clearly needed to develop it as a drug candidate for PCa.

Chen et al. (2017) reported that arenobufagin, among five bufadienolides including cinobufotalin, bufarenogin, 19-oxocinobufotalin and 19-hydroxybufalin, showed the highest potency in suppressing the progress of epithelial-mesenchymal transition (EMT) in PC-3 cells, leading to decreased ability of migration and invasion (Chen et al., 2017). Arenobufagin (8 nM) time-dependently (24, 36 and 48 h) downregulated EMT markers in PC-3 cells, including slug, zinc finger E-box binding homeobox 1 (ZEB1), snail, N-cadherin, vimentin and Twist1 as confirmed by the Western bolt experiment. In addition, β-catenin was reduced at both mRNA and expression levels by arenobufagin, which then lead to the downregulation of its downstream genes including *Met*, *LEF*, *TCF*, *c-Myc* and *cyclin D1*. These effects can be reversed by β-catenin overexpression, suggesting the network of arenobufagin with β-catenin. Arenobufagin (1 mg/kg) reduced tumor growth without altering the body weight or causing harms to major organs including heart, liver, spleen, lung and kidney. In the *in-vivo* PC-3 cells pulmonary metastases model, arenobufagin markedly reduced the number and size of tumor metastatic foci in lung tissues, suggesting its dual role in preventing tumor growth and metastasis, warranting further study (Chen et al., 2017).

Niu et al. (2018) reported the anticancer effects and the mode of action of another TVA, cinobufagin, in CRPC PC-3 cells. Cinobufagin could significantly suppress PC-3 cells proliferation, with an approximately IC_50_ of 100 nM (24 h) or 50 nM (48 h), suggesting a dose- and time-dependent manner. When tested in colony formation, cinobufagin possessed a much lower IC_50_ (slightly lower than 5 nM). Mechanistically, cinobufagin induced apoptosis of PC-3 cells *via* down-regulating anti-apoptotic MCL-1 protein (Niu and Qin, 2018). Cinobufagin appears to be much more potent than bufalin, which has an IC_50_ of 1.28 μM in PC-3 cells.

### 3.2 Combinational therapy of TVAs in PCa

In addition to its role in working alone to suppress PCa, TVA bufalin has also been found to work as a chemo-sensitizer when combined with other conventional therapeutics.

Bufalin was identified as a possible DNA topoisomerase II (Top II) inhibitor (Hashimoto et al., 1997; Pastor and Cortes, 2003). Previous *in-vitro* studies showed that sequential administration of different Top isomer inhibitors exhibited improved outcomes as compared to simultaneous administration, suggesting a feasible combinational strategy (Cho and Cho-Chung, 2003; Griffith and Kemp, 2003). Recently, Gu and Zhang (2021) investigated the combination of low-dose (0.4–0.8 mg/kg) bufalin with hydroxycamptothecin, a Top I inhibitor (Gu and Zhang, 2021). In this study, CRPC DU145 cells xenograft model in nude mice were constructed and treated by hydroxycamptothecin (2 mg/kg) combined with 0.4 mg/kg, 0.6 mg/kg or 0.8 mg/kg bufalin, respectively. The results showed that among all treatments, the combination of hydroxycamptothecin with 0.6 mg/kg bufalin showed the strongest tumor-reducing effect (93% inhibition) than the other two combinations or monotherapy, bufalin at 1 mg/kg (∼30% inhibition) or hydroxycamptothecin at 2 mg/kg (58% inhibition), without altering body weight significantly (Gu and Zhang, 2021). This combination, named as H6B, induced significantly higher apoptosis but reduced proliferating cell nuclear antigen (PCNA) proteins in the tumors than the other treatments as confirmed by the TUNEL assay and immunohistochemistry, respectively. Western blot assay showed that H6B increased pro-apoptotic proteins such as Bax, p53 and programmed cell death protein 4 (PDCD4); whereas it decreased anti-apoptotic proteins such as Bcl-XL and p-AKT (Gu and Zhang, 2021). While this study presented a possible combinational treatment that was safe and can be further validated in other models and even in humans, it remains unclear if H6B inhibit Top I/II in the treated tumor tissue.

## 4 Other therapeutic implication of TVAs in PCa

Growing evidence has suggested that TVAs may have other therapeutic application in treating PCa.

Firstly, both cinobufagin and bufalin could inhibit P-glycoprotein (P-gp, also named as ABCB1 or multidrug resistance mutation 1, MDR1) (Yuan et al., 2017; Madugula and Neerati, 2020; Zhan et al., 2020; Neerati and Munigadapa, 2022). Since P-gp plays an essential role, and sometime the leading role in inducing anticancer drug of both conventional and targeted therapies resistance *via* transporting them out of cancer cells (Robey et al., 2018; Wang et al., 2020b; Feng et al., 2020; Thomas and Tampe, 2020; Wang et al., 2021), cinobufagin and bufalin may likely have potentials in sensitizing certain conventional chemotherapeutics that are substrates of P-gp. In addition, as all of these bufadienolides possess the same pharmacophore which indicates that they may have similar bioactivities, it is reasonable to predict that other TVAs (in addition to cinobufagin and bufalin) may also impact P-gp and may have synergistic/sensitizing effects in PCa treatment when used by combination (Wu et al., 2020a; Gao et al., 2020; Chen et al., 2021), warranting further exploration. Furthermore, it’s also worth studying whether TVAs impact other members of ABC transporters. Thus, a broader screening and validation is necessary to explore their full potential.

Secondly, TVAs can induce cytochrome P450 3A in the pharmacokinetic (PK) study (Jiang et al., 2012; Dai et al., 2019), suggesting that they may affect other drugs metabolism and requiring a real-time monitor of PK profiles when used by combination.

Thirdly, since TVAs could alleviate cancer-related pain, it is meaningful in trying optimal combinational strategies with certain anticancer drugs (Xu et al., 2019).

Furthermore, there are several bufalin-derived TVAs that show better inhibitory effect in PCa cells than bufalin, including compound 6 (Figure 2), a de-hydroxyl bufalin, showed higher AR binding affinity but lower inhibition on the Na^+^/K^+^-ATPase, which may suggest a higher cytotoxic effect to PCa cells but lower toxic effect to heart, warranting further evaluation (Tian et al., 2014).

While several TVAs derivatives also exhibited promising anticancer effects in PCa and other cancer types (Yuan et al., 2014; Meng et al., 2021; Sampath et al., 2022), their application remains to be fully exploited. It’s also noteworthy that except for bufalin, arenobufagin, and cinobufagin, very few studies of bufotalin and resibufogenin in PCa have been reported in the past decade.

## 5 Toxic and potential adverse effects of TVAs

One of the major challenges in using TVAs is the toxic effect, which may significantly cripple their application potential in PCa. Thus, the toxic effects and the associated mechanism are discussed briefly.

### 5.1 Cardiac toxic effects *via* regulating Na^+^/K^+^-ATPase

Several TVAs have been confirmed to induce cardiac toxicity. Resibufogenin (0.2 mg/kg, iv) could significantly increase heart burden as indicated by contractile force in rabbit, cat and dog, leading to delayed afterdepolarization and triggered arrhythmias (Xie et al., 2001). These effects were partially mediated by its disturbance of Na^+^/K^+^-ATPase which caused calcium (Ca^+^) overload (Xie et al., 2001). Similarly, in human cardiomyocytes model, bufalin (30–300 nM) showed a biphasic effect on the contractility, which was strengthening contractility, accelerating conduction, and increasing beating rate at the earlier stage, while in the opposite when at the later stage (Li et al., 2020).

### 5.2 Neuron toxicity due to inhibit voltage-gated potassium channels

TVAs are known to cause neuron toxicity as reported previously (Brubacher et al., 1999; Dasgupta, 2003; Ma et al., 2007). In addition to the inhibition of Na^+^/K^+^-ATPase, in rat hippocampal neurons (Wang et al., 2014a), both resibufogenin and cinobufagin could also inhibit outward delayed rectifier potassium current (Hao et al., 2011), which may work together to induce toxicity in neuron system. However, we believe that more studies are needed to reveal the doses or concentrations on inducing human neuron cells related toxicity.

### 5.3 Drug-drug interactions due to the inhibition of human cytochrome P450 3A4 (CYP3A4)

A study by Li et al. (2009) found that bufalin had an inhibitory toward recombinant human CYP3A4 *in vitro*, with an IC_50_ of 14.52 μM, leading to increased elimination half-time, peak plasma level of midazolam (a substrate of CYP3A4) in the rat model. Thus, when being used with combination, adverse effects due to CYP3A4 inhibition of TVAs should be monitored and prevented.

### 5.4 The narrow therapeutic window

It’s known that in mouse model the median lethal dose (LD_50_) of bufalin in nude mice is 2.2 mg/kg (Tu et al., 2000), which is pretty close to the doses of achieving therapeutic effects of tumor inhibition (normally not more than 1 mg/kg), suggesting a very narrow therapeutic window, and that the accumulative TVAs may further worsen certain toxic effects. Thus, when being tested in humans, a close monitor of serum concentration is necessary.

## 6 Discussion and future perspectives

Cancers have become a great burden to modern people due to high prevalence and high cost in treatment and care (Desai et al., 2021; Wells, 2021). Cancer-related deaths rank the second among all deaths caused by different diseases (Siegel et al., 2022). As our major research interest, PCa stands out for three reasons, the most diagnosed cancer in men, the second most cancer deaths in men globally, and extremely high rate of drug resistance (Wade and Kyprianou, 2018). Currently, effective therapeutic strategies for PCa include surgery, cytotoxic chemotherapy agents, AR inhibitors, PARP inhibitors, and radiopharmaceuticals, etc. (Do and Webster, 2021). Unfortunately, the vast majority of PCa patients will develop acquired resistance to most of these therapeutic agents (Moreira-Silva et al., 2022).

Toad venom is a traditional Chinese medicine that has been applied (mostly used by certain extraction/mixture in combination with other drugs) in clinic for hundreds of years in China (Li et al., 2021a). It should be mentioned that all the approved drugs contain the extraction of toad venom but not the isolated active components such as these discussed bufadienolides in this review. For example, while Chansu injection, an approved drug in China for infective diseases, has been evaluated for its potential in cancer treatment, its effectiveness and safety among cancer patients are yet to be proved (Jia et al., 2022). A clinical study, published in 2016 in stage III-IV patients of non-Hodgkin lymphoma, showed that the combination of Chansu injection with EOAP (etoposide, vincristine, cytosine arabinoside and prednisone) failed to improve the therapeutic effect when compared to EOAP group (Niu et al., 2016). Another clinical evaluation showed that while Chansu injection might enhance the treatment effects of certain anticancer agents (Ma et al., 2018), we believe that further broader clinical trials are still needed to validate the efficacy.

It’s assumed that bufadienolides have several advantages over those approved drugs, because 1) bufadienolides are new therapeutic agents with distinct structures, and laboratory studies have suggested that PCa cells are sensitive to them. Thus, it’s likely that PCa cells may not be able to quickly develop resistance. 2) The above mentioned approved drugs are all single-targeted agents, which can be antagonized by adaptation of PCa cells. Bufadienolides are known for multi-targeted compounds, rendering them hard to develop resistance by PCa cells. 3) Reports have shown that P-gp can induce resistance of some PCa drugs including docetaxel (Kato et al., 2015), PARP inhibitor talazoparib (Naito et al., 2021; Teyssonneau et al., 2022), etc. As P-gp is one of bufadienolides’ targets, thus, to reverse or achieve sensitizing effects, it’s reasonable to use combinational regimens, including combination composed with approved drugs in PCa. However, cautions remain since 1) the efficacy and safety of pure isolated bufadienolides in human is unknown; 2) bufadienolides may have intensive drug-drug interactions as they have interactions with cytochrome P450 3A (Jiang et al., 2012; Dai et al., 2019); and 3) it’s unclear of the exact targets.

Though these components have been extensively studied in the past decade, none of them have been approved. The application of TVAs in PCa is still at early stage but is attracting more attentions recently.

### 6.1 Summary of TVAs in PCa

**FIGURE 3:**
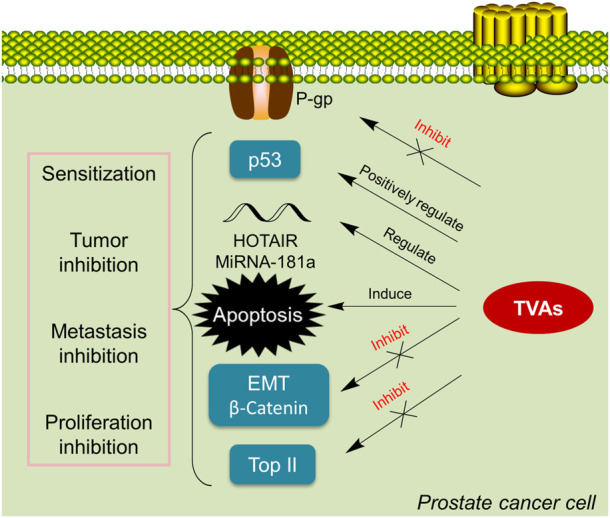
TVAs suppress cancer growth *via* various pathways. TVAs appear to be able to suppress P-gp, activate p53, and regulate critical players in EMT, inhibit Top II and modulate certain mi/lncRNA, leading to PCa cells apoptosis which thereby suppressing cancer progression.

The above literature review has summarized the application of TVAs in PCa (Table 1; Figure 3). Generally, TVAs could suppress PCa cells proliferation *via* inducing apoptosis and regulating certain miRNAs and lncRNAs; meanwhile, they also show activity in reducing PCa cells migration and invasion *in-vitro* and *in-vivo* through negatively regulating critical players involved in metastasis. It’s known that bufalin targets steroid receptor coactivators SRC-3 and SRC-1 (Wang et al., 2014b), while growing evidence suggests that bufalin is a multi-targeting or multi-functional agent, especially in the treatment of cancers.

**TABLE 1:** Key facts of bufadienolides in the treatment of PCa (as of August 2022).

TVAs	Mechanisms/Targets	Effects	Ref
Bufalin	Suppressing p53	Reducing tumor growth (1.5 mg/kg, IP)	Zhang et al. (2018)
	Regulating HOTAIR	Inhibiting PCa cells metastasis	Zhang et al. (2019b)
	Regulating miRNA181a/apoptotic proteins	Inducing PC-3 apoptosis	Zhai et al. (2013)
	Un-defined	Inhibiting DU145 cells *in-vitro* and *in-vivo*	Liu and Huang (2016)
	Inhibiting Top II and inducing apoptosis	Sensitizing hydroxycamptothecin	Gu and Zhang (2021)
Arenobufagin	Down-regulating EMT	Inhibiting PC-3 metastasis *in-vivo*	Chen et al. (2017)
Cinobufagin	Down-regulating MCL-1	Killing PC-3 cells	Niu and Qin (2018)
	Inhibiting P-gp	Sensitizing drugs that are P-gp substrates	Griffith and Kemp (2003)

Note: HOTAIR, HOX, transcript antisense RNA; EMT, epithelial-mesenchymal transition; P-gp, P-glycoprotein.

By far, except for bufalin that has been extensively studied, the therapeutic applications of other TVAs in PCa are yet to be fully revealed. While we suspect that since they all share a very similar scaffold, there may be limited differences of the underlying mechanisms, requiring further validations.

It also comes to our notice that TVAs have been approved and/or under active clinical evaluations only in China. Currently, combinational therapies of using some of TVAs are also actively tested in clinical trials, such as the combination of thalidomide with cinobufagin to treat lung cancer cachexia (Xie et al., 2018). Other TVAs-related clinical trial was either conducted more than 10 years ago (Meng et al., 2009), or using formulations made of toad venom extraction rather than isolated single component (Meng et al., 2012; Wu et al., 2020b; Tan et al., 2021). Since both the active components (major and minor) and the associated mechanisms remain largely elusive, thus, these formulations will likely meet many obstacles to be approved in other countries outside China due to different new drugs regulations. More studies using corresponding isolated pure compounds are in urgent need to support their further evaluation in humans.

### 6.2 Future perspectives

While the anticancer of TVAs in PCa can be confirmed in lab (*in-vitro* and *in-vivo*), much more works are needed before they can be eventually applied in patients worldwide. The authors propose that six future directions are worth trying.

(1) Rational design of TVAs derivatives or analogs *via* the assistance of computer-aided drug design (CADD). These structures of bufadienolides can serve as leading compounds that may undergo structural modification for improved target-binding and anticancer effects. By far, this research area is extremely undeveloped. Very few studies have been published, and most of them are focusing on the modification of hydroxyl groups at different positions (Sampath et al., 2022). Further and varied structural modification at other positions and functional groups are necessary.
(2) In-depth pharmacological/mechanistic study for target(s) identification and verification. While bufadienolides appear to regulating multifaceted signal pathways and targets in PCa, it remains elusive regarding the decisive factor. Proteomics study and gene sequence after TVAs treatment along with the associated pharmacological and validation studies is required.
(3) Following the pharmacological/mechanistic study, toxicological mechanisms, beside their inhibition on Na^+^/K^+^-ATPase or other ion channels, are needed. In addition, it is also possible that certain metabolites of TVAs may contribute to toxic effects, requiring further validation.
(4) PK study. The PK study can answer the time-course of the absorption, distribution, metabolism and elimination, as well as toxicity of bufadienolides, which may offer solutions for the doses and frequency of administration in PCa patients. Unfortunately, there is no PK data using isolated TVAs in humans. Recently in 2019, a PK study using bufalin in rats were published (Wei et al., 2019). It is shown that bufalin (10 mg/kg, oral administration) reached the peak serum concentration (14.722 ± 4.681 ng/mL) after only 15 min, which had a half time of 5.7 ± 3.06 h (Wei et al., 2019). Bufalin could quickly undergo metabolism into more than nine different metabolites. This study provided very useful information of using bufalin in rats, which may help to design and develop protocols in monitoring metabolism of TVAs in humans. In addition, these identified metabolites may help to reveal potential pharmacological effects as well as toxic effects in humans.
(5) More *in-vivo* models validation of bufadienolides in PCa. In addition to *in-vitro* models, *in-vivo* models including patient-derived xenograft models are warranted. Furthermore, due to its innate toxic effects, the combinational regimens of low-dose bufadienolides with certain conventional chemotherapeutics will be promising.
(6) Deciphering associated resistance reasons and developing combinational strategy. Drug resistance is a major obstacle in PCa treatment (Do and Webster, 2021; Zhao et al., 2021). While we suspect that PCa cells may not develop resistance to bufadienolides easily, it’s largely unknown when and how, as well as the resistance rate and resistant mechanisms. For the full application and indications, more studies are needed to reveal resistant reasons.

Finally, more clinical trials in PCa are necessary to test the efficacies of TVAs including their pharmaceutical formulations.

## 7 Conclusion

PCa, due to its high incidence rate and prevalence of drug resistance, is one of the leading threats to men’s health. Chinese traditional medicine toad venom and TVAs have emerged as promising therapeutic agents in PCa, which have been validated by cell- and animal-based models. Further in-depth studies are also clearly needed for the underlying mechanisms, toxicology, and for exploring combinational therapies in PCa.
